# Editorial: Machine Learning for Big Data Analysis: Applications in Plant Breeding and Genomics

**DOI:** 10.3389/fgene.2022.916462

**Published:** 2022-05-31

**Authors:** Salvatore Esposito, Valentino Ruggieri, Pasquale Tripodi

**Affiliations:** ^1^ CREA Research Centre for Cereal and Industrial Crops, Foggia, Italy; ^2^ Biomeets Consulting, ITNIG, Carrer d'Àlaba, Barcelona, Spain; ^3^ CREA Research Centre for Vegetable and Ornamental Crops, Pontecagnano Faiano, Italy

**Keywords:** machine learning—ML, genomics, phenotyping algorithms, high throughput (HTP), genotyping

**FIGURE 1:**
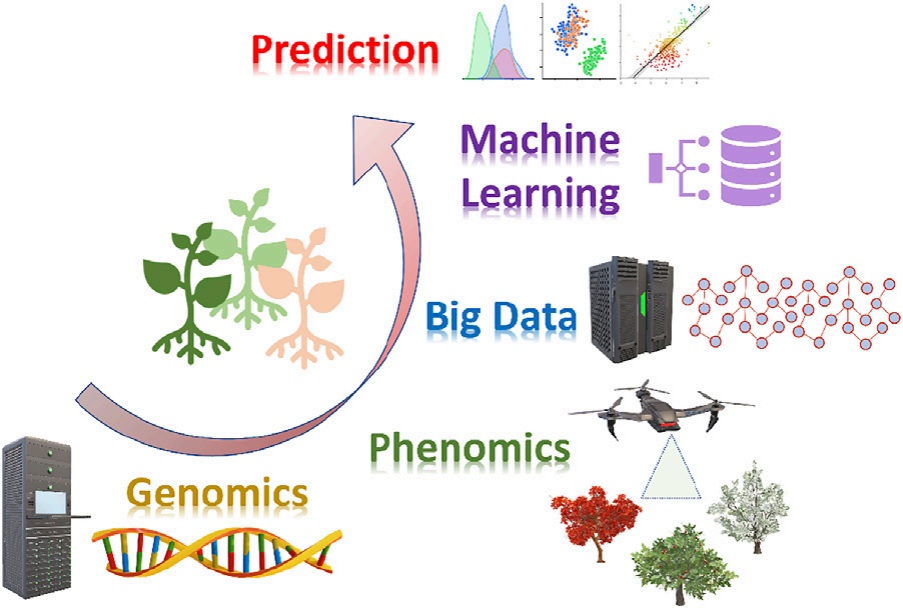
Machine learning and its application for big data analysis in plant breeding. Cutting edge genomics and phenomics technologies generate multi-omics datasets to be analyzed through machine learning approaches toward management and predictive data analytics.

Next-generation sequencing (NGS) technologies, advanced phenotyping platforms, and machine-learning (ML) as “the science of programming computers so they can learn from data” are leading a new revolution in plant breeding, facilitating a deep understanding of the genotype and its relationship with the phenotype, especially for complex traits (Figure 1).

In this stimulating scenario, the Research Topic introduces five original research papers and one review focusing on computational analysis and machine learning-based approaches for plant breeding and genomics. Among the possible strategies that researchers can adopt to accelerate crop breeding and boost plant production, genomic selection (GS) was proposed in the last few years to design novel breeding programs and to develop new markers-based models for genetic evaluation, thus providing new opportunities to increase the genetic gain of complex traits per unit time and cost. However, as summarized in the review by Danilevicz et al., most of the tools routinely used in genomic selection studies are not designed to capture non-linear relationships within multi-dimensional datasets or deal with big datasets such as imagery collected by drones. By contrast, given the capacity to extract data features and represent their relationships at multiple levels of abstraction, ML algorithms have the potential to overcome the barriers in prediction accuracy occurring in the tools routinely used for genotype to phenotype predictions. There are three ways to classify machine learning methods, including supervised and supervised models, linear and nonlinear algorithms, and shallow and deep learning models. Artificial neural networks (ANNs), deep neural networks (DNNs), convolutional neural networks (CNNs), random forest (RF), and support vector machines (SVMs) are only a few examples of nonlinear nonparametric machine learning algorithms, that can be applied for processing non-linear data in plant studies. The review by Danilevicz and colleagues summarized the challenges of applying different machine learning methods for increasing the accuracy in predicting phenotypic traits based on molecular markers, environment data, and imagery. The paper by Montesinos-López et al. also underlies the need to overcome the recent limits of genomic selection and proposed a new deep-learning calibration method that can enhance genome-based prediction of continuous crop traits. One of the big challenges in GS studies is the training process, mainly due to the high number of hyper-parameters that must be tuned, thus increasing the probability to add bias in the analysis. For these reasons, the authors proposed a simple method for calibrating (adjusting) continuous predictions resulting from deep learning applications. The proposed deep learning calibration method (DL_M2) was tested in four different crop breeding datasets and its performance was compared with the standard deep learning method (DL_M1) and with the standard genomic best linear unbiased predictor (GBLUP). The authors claimed that, although the GBLUP was the most accurate model, the proposed deep learning calibration method (DL_M2) helped to increase the genome-enabled prediction performance in all datasets.

In terms of practical applications, there are many opportunities to put state-of-the-art techniques in machine learning models. For example, ML can also be used to improve the accuracy of the plant phenotyping process, and the predicted data can then be used in turn for QTL (quantitative trait loci) studies. Traditional phenotyping methods are usually labor-intensive, time-consuming, and prone to errors, whereas high-throughput phenotyping platforms can effectively attain physiological traits related to photosynthesis and secondary metabolites that can enhance breeding efficiency. Kumar et al. evaluated supervised machine learning models for their accuracy in distinguishing water-stressed plants and identifying the most important water stress-related parameters in lettuce. The authors reported that random forest (RF) had an accuracy of 89.7% using kinetic chlorophyll fluorescence parameters, whereas the neural network (NN) reached an accuracy of 89.8% using hyperspectral imaging-derived vegetation indices. Then, the top 10 parameters selected by RF and NN were genetically mapped using the *Lactuca sativa* × *L. serriola* interspecific recombinant inbred line (RIL) population, allowing the identification of 25 QTL segregating for water stress-related traits, 26 for the chlorophyll fluorescence traits, and 34 for spectral vegetation indices (VI). Shin and Nuzhdin also adopted random forest models to apply the samples prioritization scheme, revealing how ML facilitated the investigation of predictive causal markers in most of the biological scenarios simulated in the present study.

Besides QTL mapping and GS studies, this Research Topic also covered the power of ML in predicting regulatory sequences involved in stress tolerance mechanisms. In this context, Gupta et al. developed the gene regulation and association network (GRAiN) for rice (*Oryza sativa*). GRAiN is an interactive query-based web-platform built by applying a combination of different network inference algorithms to publicly available gene expression data. The supervised machine learning framework can convert intricate network connectivity patterns of transcription factors (TFs) into a single drought score, allowing the prediction and the validation of *OsbHLH148* as an important player involved in rice drought stress.

Computational algorithms can be successfully applied for the analysis of big data generated from cutting-edge NGS platforms. Niu et al. reported the *de novo* assembly of a macadamia tree by a combination of Oxford nanopore and Hi-C (high-throughput chromosome conformation capture) sequencing technologies. Although no ML model was reported by authors, the extensive analyses performed shed light on the genome evolution of this species providing experimental support for detecting genes underlying the biosynthesis of unsaturated fatty acids, thus laying the basis of genomic-assisted breeding for this species. In conclusion, the papers collected in this Research Topic have presented some of the recent advances in the application of machine learning models to different omics disciplines, enhancing their integration toward a resolution of key biological questions. For this purpose, “explainable machine learning” will be a key area for genotype to phenotype research, especially in generating accurate predictions combined with reliable interpretations, as also reported by Danilevicz et al.

